# Lymph node ratio is a prognostic factor for non-small cell lung cancer

**DOI:** 10.18632/oncotarget.5669

**Published:** 2015-09-15

**Authors:** Guangyuan Sun, Lei Xue, Mingdong Wang, Xuewei Zhao

**Affiliations:** ^1^ Department of Thoracic and Cardiovascular Surgery, Changzheng Hospital, the Second Military Medical University, Shanghai, China

**Keywords:** non-small cell lung cancer, lymph node ratio, association, meta-analysis

## Abstract

The lymph node ratio (LNR) is defined as the number of pathologically positive LNs divided by the number of LNs examined. Some studies reported that high LNR was significantly associated with poor survival of non-small cell lung cancer (NSCLC). However, other studies could not confirm this result. PubMed, Embase, and the Cochrane Register of Controlled Trials were searched for relevant studies published up to July 2015. Primary outcome was the relationship between LNR and disease-specific survival (DSS) and overall survival (OS). Twelve studies with 25138 NSCLC patients were included in this meta-analysis. Higher LNR was significantly associated with decreased OS (HR = 1.93; 95% CI 1.64 – 2.28; *P* < 0.00001) and DSS (HR = 1.82; 95% CI 1.55 – 2.14; *P* < 0.00001). In the subgroup analysis, N1 stage NSCLC patients with higher LNR also showed decreased OS (HR = 1.60; 95% CI 1.25 – 2.28; *P* = 0.0002) and DSS (HR = 1.82; 95% CI 1.55 – 2.21; *P* < 0.0001). This meta-analysis indicated that LNR was an independent predictor of survival in patients with NSCLC.

## INTRODUCTION

The long-term survival of non-small cell lung cancer (NSCLC) after surgical resection remains low due to the complex biological characteristics and high recurrence and metastasis [1]. Five-year survival rates for surgically resectable NSCLC are still unsatisfactory and range from 19% for stage IIIA to 63% for stage IA [2]. Therefore, the identification of predictive and/or prognostic markers is important to stratify patients with resected NSCLC and select high-risk patients who should receive aggressive adjuvant chemotherapy.

The lymph node ratio (LNR), defined as the number of pathologically positive LNs divided by the number of LNs examined, has been reported as a useful prognostic metric. LNR incorporates both the number of pathologically positive LNs and the number of LNs examined. Some studies reported that high LNR was significantly associated with poor survival. However, other studies could not confirm this result [3-14]. Thus, the result is still controversial. We performed this meta-analysis to assess the relationship between LNR and prognosis of NSCLC.

## RESULTS

### Eligible studies

**Figure 1:**
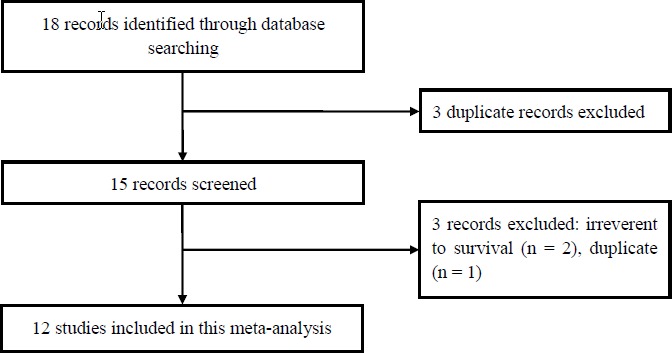
The selection of included studies

The process of identifying studies is shown in Figure 1. A total of 18 publications were identified in the initial search. Based on screening of titles or abstracts, 3 records were excluded. Full text articles were retrieved only for 15 publications and assessed for eligibility. Of these 15 publications, 3 publications were excluded. Finally, 12 studies were included in this meta-analysis.

Twelve studies with 25138 NSCLC patients were included in this meta-analysis. These studies were conducted ranged from 2011 to 2015. The median follow-up duration ranged from 1.8 to 17 years. The quality score ranged from 6 to 9. The characteristics of included studies were showed in Table 1.

**Table 1:** Characteristics of the studies included in this meta-analysis

First author	Year	Age (years)	T stage	N stage	Case number (n)	Follow-up duration (years)	Disease-specific survival reported	Overall survival reported	Adjust	Scores
Jonnalagadda	2011	<65	T1-T3	N1	4004	15	Yes	Yes	age, sex, race, histology, tumor site and status, type of surgery, and receipt of radiation therapy	8
Wisnivesky	2011	>65	T1-T3	N1	1682	15	Yes	Yes	age, sex, race/ethnicity, marital status, estimated income, comorbidities, histology, tumour status and location, type of surgery and use of chemotherapy or radiation therapy.	8
Matsuguma	2012	NA	T1-T3	N0-N2	651	17	No	Yes	age, gender, histology, pathological T status, surgical type, postoperative chemotherapy, and postoperative radiotherapy.	7
Nwogu	2012	NA	NA	N0-N2	5012	4	Yes	Yes	age, race, sex, tumor size, and histologic grade of the tumor	8
Wang	2011	31-78	T1-T3	N1-N2	301	10	Yes	Yes	Histologic type, stage, smoking, No. of involved nodes	7
Li	2013	64	T1-T3	NA	206	5	No	Yes	different nodal involvement pattern, the ratio of the number of positive LNs to the total number of LNs removed, number of LNs involved, patient age, sex, history of smoking, pathologic type, type of resection, VPI, lymph vascular invasion, and tumor size	6
Qiu	2013	59	T1-T3	N0-N2	480	3	Yes	Yes	age, sex, smoking status, location of tumor, histology, pathology T stage, pathology N stage, surgical procedure, chemotherapy, metastatic lymph node	6
Taylor	2013	NA	NA	N1-N2	1143	3.7	Yes	No	age, sex, use of preoperative radiation, use of preoperative chemotherapy, preoperative ECOG status, pathologic tumor stage, N stage, T stage, total lymph nodes removed	7
Urban	2013	66	T1-T4	N1-N2	11324	1.8	No	Yes	age, sex, race, grade, histology, laterality, surgery, pathology T stage, pathology N stage	8
Hsieh	2014	60.2	T0-T3	N2	108	2.4	Yes	Yes	T stage, Angiolymphatic invasion, Perineural invasion, operation	6
Wu	2014	34-83	T1-T3	N1	75	5.5	Yes	Yes	Sex, smoking, adjuvant therapy, angiolymphatic invasion, histology	6
Renaud	2015	58.5	T1-T4	N0-N2	152	2.7	No	Yes	Adjuvant treatment, Extracapsular spread, pathology T stage, pathology N stage, Type of surgery, Charlson comorbidity index	7

NA, not available.

### Quantitative data synthesis

**Figure 2:**
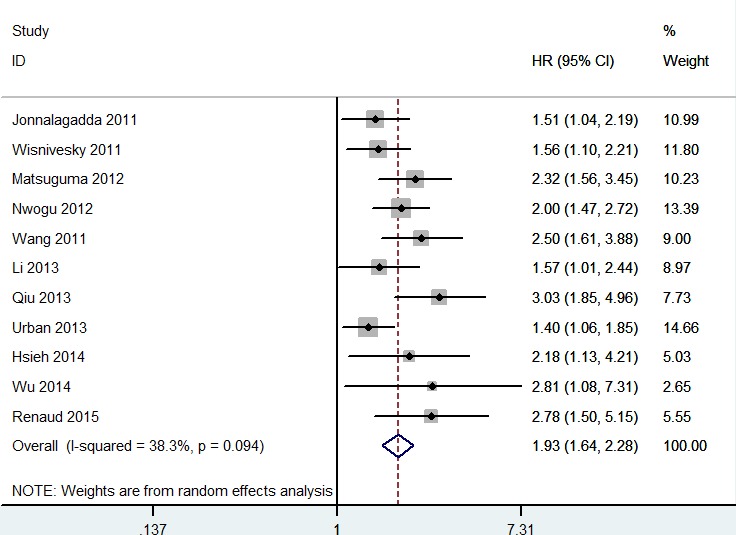
Meta-analysis of the association between LNR and OS

**Figure 3:**
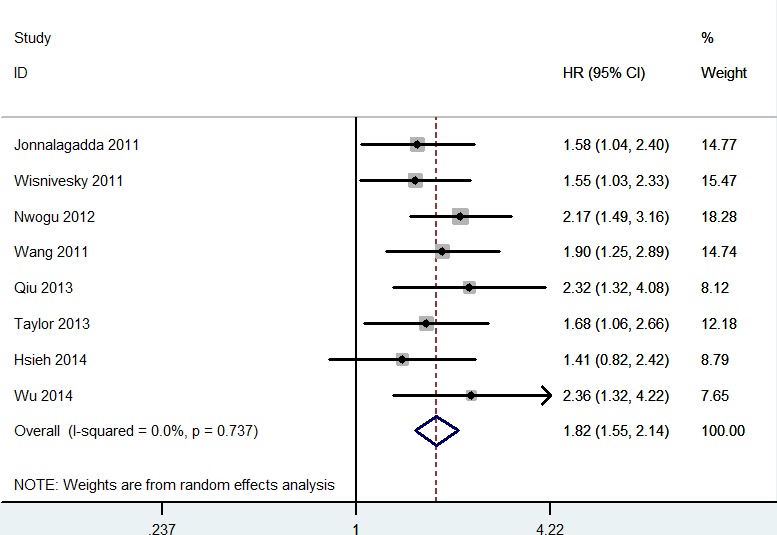
Meta-analysis of the association between LNR and DSS

As shown in Figure 2, higher LNR was significantly associated with decreased OS (HR = 1.93; 95% CI 1.64 - 2.28; *P* < 0.00001). In the subgroup analysis, N1 stage NSCLC patients with higher LNR was also associated with decreased OS (HR = 1.60; 95% CI 1.25 - 2.28; *P* = 0.0002). As shown in Figure 3, higher LNR was significantly associated with decreased DSS (HR = 1.82; 95% CI 1.55 - 2.14; *P* < 0.00001). In the subgroup analysis, N1 stage NSCLC patients with higher LNR was also associated with decreased DSS (HR = 1.82; 95% CI 1.55 - 2.21; *P* < 0.0001). In the subgroup analysis by sample size, both studies with large scale and small scale showed similar results (Table 2).

**Table 2:** Results of the meta-analysis

	No. of studies	Test of association			Model	Heterogeneity		
		HR (95% CI)	*Z*	*P* Value		*χ*^2^	*P* Value	*I*^2^ (%)
Overall survival	11	1.93 (1.64-2.28)	7.83	<0.00001	R	16.22	0.09	38.0
N1	3	1.60 (1.25-2.05)	3.73	0.0002	R	1.45	0.49	0.0
Large scale (n>1000)	4	1.60 (1.36-1.88)	5.73	<0.00001	R	3.01	0.39	0.0
Small scale (n<1000)	7	2.32 (1.91-2.82)	8.55	<0.00001	R	4.76	0.57	0.0
Disease-specific survival	8	1.82 (1.55-2.14)	7.31	<0.00001	R	4.36	0.74	0.0
N1	3	1.70 (1.31-2.21)	3.98	<0.0001	R	1.54	0.46	0.0
Large scale (n>1000)	4	1.75 (1.42-2.15)	5.32	<0.00001	R	1.86	0.60	0.0
Small scale (n<1000)	4	1.93 (1.50-2.50)	5.04	<0.00001	R	2.16	0.54	0.0

R, random-effects model.

**Figure 4:**
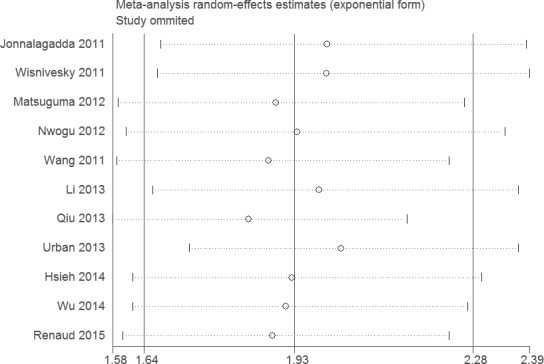
Sensitivity analysis of the association between LNR and OS

**Figure 5:**
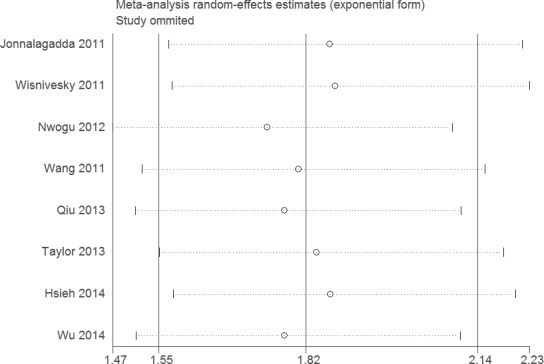
Sensitivity analysis of the association between LNR and DSS

**Figure 6:**
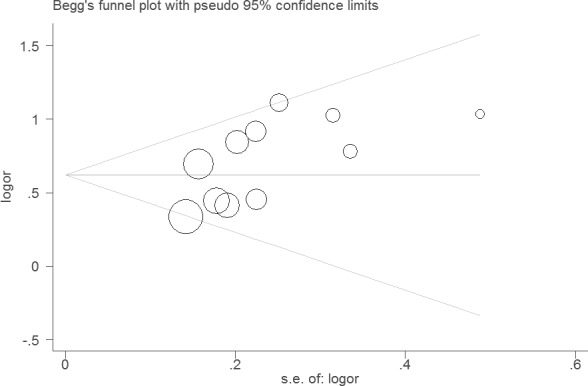
Funnel plot of the association between LNR and OS

**Figure 7:**
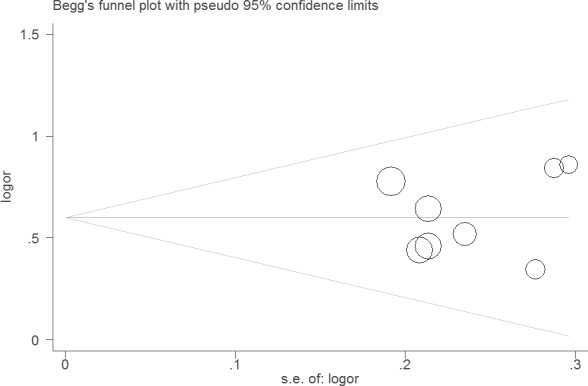
Funnel plot of the association between LNR and DSS

Sensitivity analysis did not change the results of this meta-analysis (Figures 4 and 5). The funnel plot and Egger's test were performed for the overall comparison. No obvious visual asymmetry was observed in funnel plots (Figures 6 and 7), and the *P* values of the Egger's test were greater than 0.05.

## DISCUSSION

This meta-analysis of 12 observational studies showed that NSCLC patients with higher LNR had worse outcome. NSCLC patients with N1 stage showed similar results.

The studies suggested that LNR could be a prognostic factor in other cancers. For example, Chen et al. showed that a staging system based on LNR may have better prognostic stratification of patients with esophageal squamous cell carcinoma than the current TNM system [15]. Liao et al. indicated that LNR was an important prognostic factor with regard to OS for patients with node-positive breast cancer [16]. Kutlu and colleagues suggested that LNR is an effective tool to predict survival in a western gastric cancer patient population [17]. Zhan and colleagues indicated that LNR was an independent adverse prognostic factor for pancreatic cancer [18]. In addition, Fleming et al. suggested that LNR appears to be a useful tool to identify patients with worse prognosis in node-positive early stage cervical cancer [19].

Recent studies also investigated whether patients with high LNR should receive radiotherapy. Urban et al. indicated that the survival benefit associated with postoperative radiotherapy seemed to be limited to patients with high LNR [11]. However, whether patients with high LNR should receive adjuvant chemotherapy is needed to be investigated in the future.

Our study had some advantages. First, this is the first meta-analysis about LNR and outcome of NSCLC. Second, the statistic power is well enough for this meta-analysis. However, this study also had several limitations. First, all the included studies were observational retrospective investigations. Second, lacking of the original data of the eligible studies limited the evaluation of the effects of LNR on different populations, such as smoker and non-smoker, adenocarcinoma and squamous cell carcinoma. Third, although we performed an extensive review of the main electronic databases, we cannot be sure to have included all relevant studies.

In conclusion, this meta-analysis indicated that LNR was an independent predictor of survival in patients with NSCLC.

## MATERIALS AND METHODS

### Publication search

PubMed, Embase, and the Cochrane Register of Controlled Trials were searched for relevant studies published up to July 2015. The following terms were used: (“NSCLC” or “lung cancer” or “non-small cell lung cancer”) and (“lymph node ratio” or “LNR”). The MeSH terms were used: “Lung Neoplasms” or “Carcinoma, Non-Small-Cell Lung”. No language restrictions were imposed. References from relevant articles, including review papers, were also reviewed.

### Selection and exclusion criteria

Studies were included in the meta-analysis if they fulfilled the following inclusion criteria: 1) study design: cohort studies; 2) population: patients with NSCLC; 3) primary outcome: the relationship between LNR and disease-specific survival (DSS) and overall survival (OS). Abstract, case reports, review articles, experimental studies and commentary articles were excluded. Eighteen studies were searched from PubMed, Embase, and the Cochrane Register of Controlled Trials. However, four duplicate studies and two studies without of survival were excluded. At last, twelve studies were selected for this meta-analysis.

### Data extraction

Two investigators reviewed and extracted information independently from selected publications in accordance with the above mentioned inclusion and exclusion criteria. Any conflicts over study/data inclusion were settled by a discussion between the investigators. The following items were extracted from each study if available: first author, year of publication, age, T stage, N stage, sample size, follow-up years, and covariates.

### Qualitative assessment

Two authors completed the quality assessment independently. The Newcastle-Ottawa Scale (NOS) was used to evaluate the methodological quality, which scored studies by the selection of the study groups, the comparability of the groups, and the ascertainment of the outcome of interest. We considered a study awarded 0-3, 4-6, or 7-9 as a low-, moderate-, or high-quality study, respectively.

### Statistical analysis

We estimated the hazard ratio (HR) with 95% confidence interval (CI) for primary outcome. The multivariable-adjusted HRs with 95% CIs were pooled in this analysis. Heterogeneity of the combined studies was assessed with Cochran's Q-statistic test and I^2^ test. The P value of Cochran's Q-statistic of below 0.05, was considered statistically significant heterogeneity. The I^2^ test provides a measure of the degree of heterogeneity in the results. Typically, values of 0∼25% are considered to represent no heterogeneity, 25∼50% to modest heterogeneity, 50∼75% to large heterogeneity and 75∼100% to extreme heterogeneity. A random effects model was applied. Subgroup analysis and sensitivity analysis were performed. Sensitivity analyses were conducted to assess the strength of our findings by excluding one study at a time. Publication bias was investigated by Egger's test. All statistical analyses were performed with STATA 11.0 software.
